# Planar liquid crystal optics for simultaneously surface displaying and diffraction-limited focusing

**DOI:** 10.1515/nanoph-2022-0410

**Published:** 2022-08-30

**Authors:** Zhenglong Shao, Xin Xie, Yingjie Zhou, Xiaohu Zhang, Wenjuan Du, Fan Fan, Dongliang Tang

**Affiliations:** Key Laboratory for Micro/Nano Optoelectronic Devices of Ministry of Education & Hunan Provincial Key Laboratory of Low-Dimensional Structural Physics and Devices, School of Physics and Electronics, Hunan University, Changsha 410082, China; Key Laboratory of Light Field Manipulation and Information Acquisition, Ministry of Industry and Information Technology, and Shaanxi Key Laboratory of Optical Information Technology, School of Physical Science and Technology, Northwestern Polytechnical University, Xi’An 710129, China; Key Laboratory of Optoelectronic Technology and Systems of the Education Ministry of China, Chongqing University, Chongqing 400044, China; School of Physics and Optoelectronics, Xiangtan University, Xiangtan 411105, China

**Keywords:** diffraction-limited imaging, liquid crystal, multifunctionality, surface displaying

## Abstract

Planar optical elements have attracted widespread attentions because of their precise light modulation. Liquid crystals (LCs) are well known for their applications in the current displaying field, and show great potential in planar optical elements with the development and innovation of LC micro-operation technology. However, previous researches on LC elements mainly involved only one type of optical manipulation, which inevitably limited the functional diversity. In this work, we propose a multifunctional LC element which integrates the surface display into a binary-phase focusing lens by controlling the complex amplitude of the incident light. The light modulation of the anisotropic LC molecule satisfies a sinusoidal variation, which can be regarded as the combination of a continuous amplitude modulation and a binary phase modulation. The element with millimeter size is then fabricated, and the experimental measurements agree well with our design with a high-definition surface pattern and high-quality optical focusing/imaging performance. Furthermore, as the complex amplitude modulation changes from sine to cosine function after rotating the sample by 45°, a bifocal lens with two different focal lengths is also demonstrated. We expect the proposed multifunctional LC elements can find applications in information multiplexing, image displaying, etc.

## 1 Introduction

The control of light has always played an important role in both science and our daily life [1]. For example, a lens, shaping the incident phase profile and focusing light into a tiny spot, has been widely used in our daily life, such as the camera of a smartphone and microscope objective. To correct the chromatic aberration and extend field of view, a combination of multiple curved lenses should be appropriately considered and arranged; thereby commercial achromatic lenses or wide-angle lenses are developed. Typically, these refractive optical elements rely on the phase accumulation along light propagation to realize light bending [2–4]. Therefore, it is challenging to create a compact, low-cost, and lightweight device with conventional refractive optics, and a large volume and heavy weight of the optical system is inevitable [5, 6].

In recent years, planar functional elements based on liquid crystals (LCs) have attracted significant interest benefitting from the Pancharatnam–Berry (PB) geometric phase control [7–18]. Different from the conventional phase accumulation, the PB-phase-based LC element introduces abrupt phase change through rotating the anisotropic LC molecule. Due to the self-assembly property and the easy LC alignment controlled by an external field, LC element can be simply manufactured through directly spin-coated with LC on the substrate with a predefined molecular arrangement pattern, and LC structuring techniques, such as micro-rubbing [19–21], holography [22, 23], and photo-alignment [24–27], have been successfully developed to generate the predefined molecular arrangement pattern. Therefore, a series of LC functional elements were proposed and experimentally demonstrated, such as focusing lenses [8, 11, 17, 28], polarization gratings [12, 13] and vortex beam generators [14, 15, 29]. However, the mentioned explorations of LC functional elements are mostly focused on achieving a single function, which greatly limits the multifunctionality development of LC elements.

In this paper, we utilize the complex amplitude modulation existing in the anisotropic LC molecule to construct a multifunctional LC element which integrates a surface displaying performance into a binary-phase focusing lens. The light modulation of the anisotropic LC molecule follows a sinusoidal variation, which can be regarded as the combination between a continuous amplitude modulation and a binary phase modulation in mathematic. Therefore, the surface displaying property and binary-phase focusing lens can be effectively combined through arranging the orientation angles of LC molecules. Since the light modulation is solely dependent on the orientation of the LC molecule, our element exhibits broadband performance and works well at other working wavelengths. Furthermore, a spatial multiplexing LC element is designed and demonstrated to generate a bifocal lens with two different focal lengths through rotating the sample with 45°, at the same time, two complementary near-field patterns can be observed. We expect that the proposed LC elements with simple design, mature process and low cost can find promising applications in the fields of information encoding, optical data storage, and other related optical fields.

## 2 Results

### 2.1 Complex amplitude modulation by LC and element design

**Figure 1:**
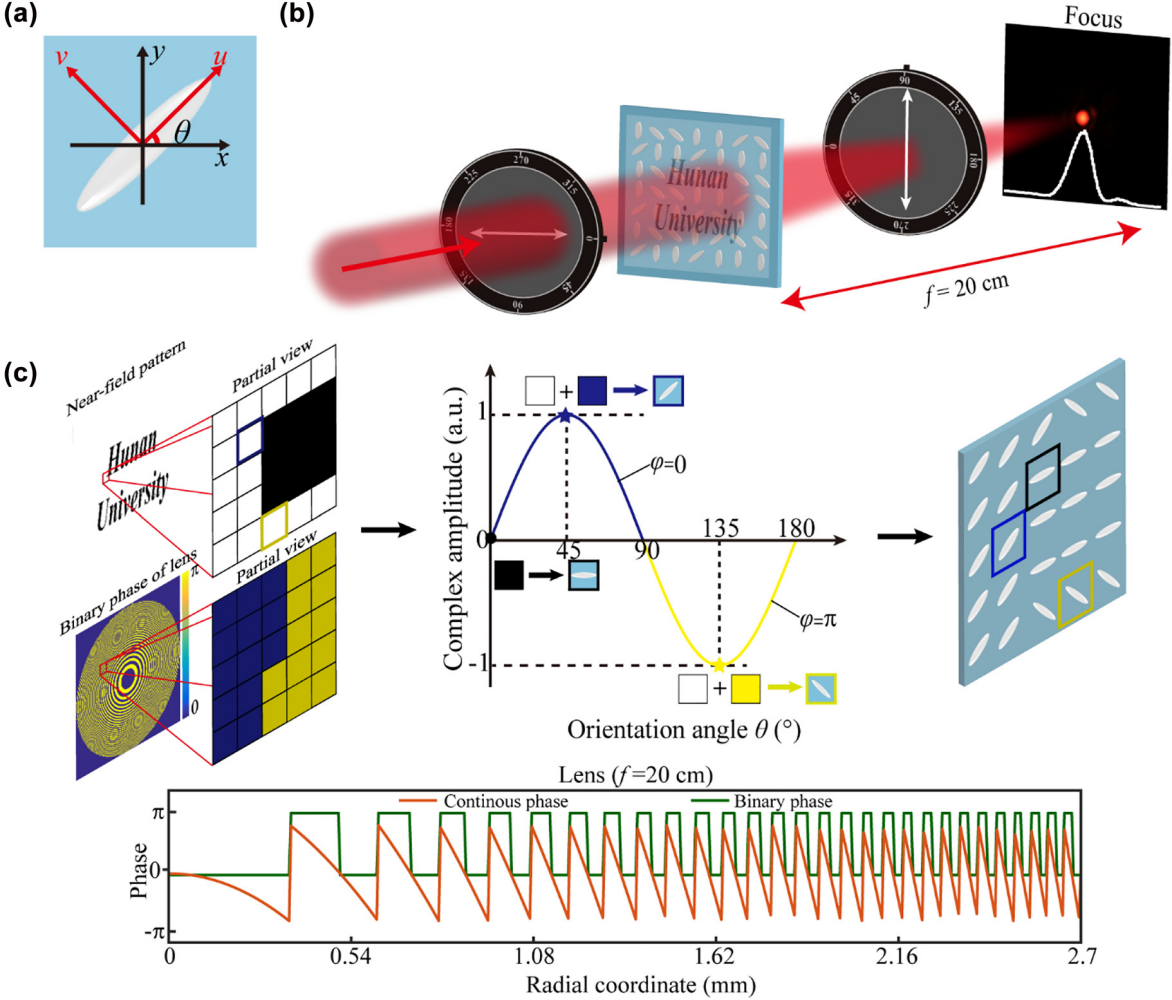
Schematic diagram and design method of the multifunctional LC element for simultaneously surface displaying and far-field focusing. (a) Schematic of the anisotropic LC molecule, where *θ* is the orientation angle of the LC molecule with respect to the *x*-axis. (b) Schematic illustration of the multifunctional LC element for simultaneously surface displaying and far-field focusing. The polarization state of the analyzer is orthogonal to that of the polarizer. (c) Design mothed of the multifunctional LC element. Normalized complex amplitude of the cross-polarized transmitted light has the same curve with the relation of sin(2*θ*). Phase part of the blue curve is 0 while that of the yellow curve is *π*. By combining the target near-field pattern and the binary-phase distribution, the LC orientations can be determined based on the curve and the multifunctional LC element is then constructed.

When light passes through an anisotropic LC molecule, the light modulation can be derived from the Jones matrix. Assuming that the main axes of LC molecule in local *u–v* coordinates, the Jones matrix can be written as

(1) $$J_{uv}=\left[\begin{array}{cc} t_{u} & 0\\ 0 & t_{v} \end{array}\right]$$

where *t*_*u*_ and *t*_*v*_ are the transmission coefficients along the local *u–v* axes, which rotate an angle of *θ* from *x–y* coordinate, as shown in Figure 1(a). Therefore, the matrix in the *x–y* coordinate can be expressed as

(2) $$\begin{array}{ll} J_{xy} & =R\left(\theta \right)J_{uv}R\left(-\theta \right)\\ & =\left[\begin{array}{cc} \cos\theta & -\sin\theta \\ \sin\theta & \cos\theta \end{array}\right]\left[\begin{array}{cc} t_{u} & 0\\ 0 & t_{v} \end{array}\right]\left[\begin{array}{cc} \cos\theta & \sin\theta \\ -\sin\theta & \cos\theta \end{array}\right] \end{array}$$

When a linear polarized light along *x*-axis illuminates the LC molecule, the output light can be calculated as:

(3) $$\left[\begin{array}{c} E_{\text{xout}}\\ E_{\text{yout}} \end{array}\right]=J_{xy}\left[\begin{array}{c} E_{\text{xin}}\\ E_{\text{yin}} \end{array}\right]=J_{xy}\left[\begin{array}{c} 1\\ 0 \end{array}\right]=\left[\begin{array}{c} t_{u}cos^{2}\theta +t_{v}\sin^{2}\theta \\ \left(t_{u}-t_{v}\right)\sin\theta \cos\theta \end{array}\right]$$

According to Eq. (3), we can find that the output light field contains the cross-polarized component of *E*_yout_, which satisfies the relationship of sin(2*θ*): a continuous amplitude changes from 0 to 1 can be obtained when the orientation *θ* varies from 0° to 180°, and the phase remains unchanged when the orientation *θ* changes from 0 to 90° or 90° to 180°, but there is an abrupt phase shift of *π* as *θ* is 90°. Therefore, two different operation functions, i.e., continuous amplitude control and binary phase modulation, can be realized simultaneously through only one LC element with an elaborate LC arrangement. As for experimental tests, if the incident light sequentially passes through a linear polarizer, our LC element and an analyzer (the polarization state of the analyzer is orthogonal to that of the polarizer), and the LC element is rotated to a suitable angle, one can observe a pattern at the sample surface and a near-diffraction-limited spot at the preset focal plane, as shown in Figure 1(b).

Figure 1(c) presents the detail of the orientation determination of each LC molecule: (a) we calculate the candidate orientation angle *θ* of each LC molecule by the target near-field pattern and *I =* sin^2^2*θ*; (b) the phase distribution of a focusing lens should be binarized because the LC molecule only provides a binary phase; (c) the appropriate orientation angle of each LC molecule is selected from the candidate orientation angles by considering the binary phase distribution. For example, to achieve the intensity value of 1 in near-field pattern, we can choose the orientation angle as 45° or 135° but the phase shift between these two orientations is *π*. As for the intensity value of 0 at near-field pattern, we choose 0° as the orientation angle. Therefore, by arranging the orientation of each LC molecule, both intensity control and binary-phase modulation can be satisfied.

### 2.2 Experimental demonstrations

The LC element is then fabricated based on a standard photoalignment technology through using a digital micro-mirror device (DMD) (the details are provided in Section S1, Supporting Information). It contains 1000 × 1000 pixels and the dimensions is 5.4 × 5.4 mm (pixel size is 5.4 μm). The target pattern at the sample surface is the image containing “*Hunan University*” (other samples with different near-field patterns are also designed in Figure S2), and the focal length of the element is set as 20 cm when the sample works as a focusing lens at the wavelength of 638 nm. A home-built optical experimental setup is used to characterize the performance of our dual-functional LC element. A parallel light is generated by the combination of a laser and a collimator. The sample is inset into an orthogonal-polarization optical path (the polarization state of the analyzer is orthogonal to that of the linear polarizer) and mounted on a three-dimensional translation platform, which allows the adjusting of the sample. In near-field tests, a CCD with a magnified lens is used to capture the pattern at the sample surface. In the focusing tests, the magnified lens is removed and the focusing spot is captured directly by the CCD.

**Figure 2:**
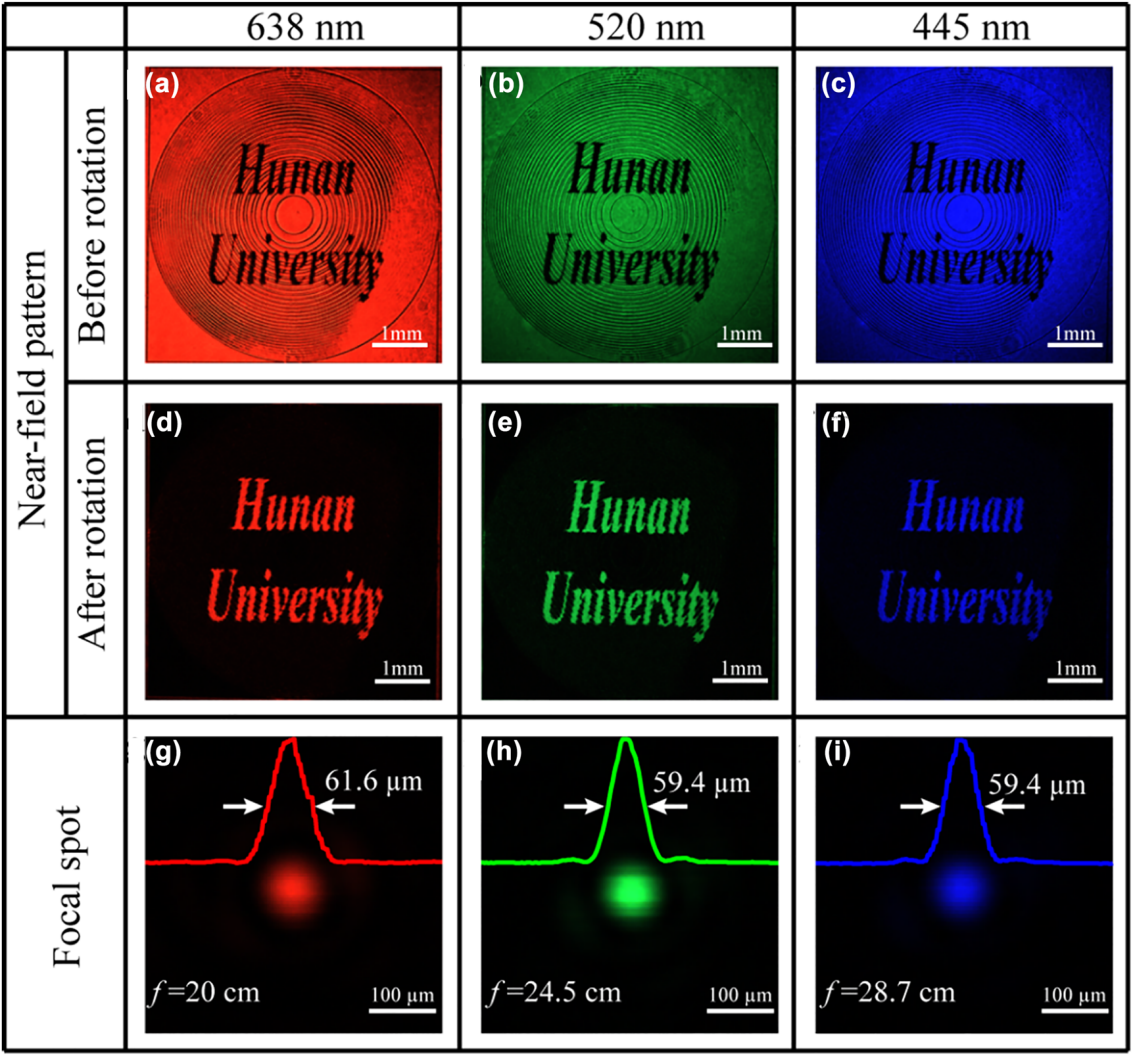
Experimental results of the multifunctional LC element. (a–c) Near-field patterns by rotating the sample at a suitable angle (*φ*) for wavelengths of 638 nm, 520 and 445 nm, respectively. (d–f) Corresponding complementary near-field patterns by further rotating the sample with 45° (i.e., *φ* + 45°). (g–i) Light distributions at corresponding focal planes when the sample rotation angle is *φ*. The existed chromatic aberration causes the shifted focus, which moves from the preset 20 cm at 638 nm to 24.5 cm at 520 nm and 28.7 cm at 445 nm.

Figure 2(a) shows the result of the near-field experiment at the wavelength of 638 nm by rotating the sample at an appropriate angle in the orthogonal-polarization optical path. The near-field pattern will switch to its complementary image when the sample is further rotated 45°. This phenomenon is consistent with the derivation in Eq. (3), where if the orientation angle changes from *θ* to *θ* + 45°, the normalized intensity of the output light changes from sin^2^2*θ* to cos^2^2*θ*, thereby a complementary image can be observed. The complementary image in Figure 2(d) not only provides a clear visual effect but also increases the flexibility of the proposed LC element as we can choose two near-field states at will. To characterize the focusing property of the element, we measure the intensity profile at the focal plane. As shown in Eq. (3), it is easily concluded that the far-field performance coming from the binary-phase modulation would be affected because of the near-field pattern. To observe the influence, an iris with an adjustable diameter from 2 to 5 mm is placed at the front of the LC sample and the focusing performances are presented in Section S3, Supporting Information. In comparison, a binary-phase focusing lens without the near-field pattern is also fabricated and tested. We can find that the near-field pattern would affect the shape of the focus and the circular symmetry can be acceptable when the iris diameter is 2 mm. Therefore, an iris with the diameter of 2 mm is inset before our LC element for a better focusing and imaging behavior. Figure 2(g) shows the measured focal spot at the wavelength of 638 nm, and the full-width at half maximum (FWHM) of the focus is 61.6 μm, approaching the diffraction-limited spot size of 63.8 μm, calculated by the formula of 0.5*λ*/NA.

As the light modulation is solely dependent on the orientation angle of the LC molecule without considering the material dispersion, as shown in Eq. (3), our LC element can work well under other incident wavelengths. The near-field patterns and the focusing spots can be observed under the illumination of the green (520 nm) and blue light (445 nm). The focal plane shifts to 24.5 and 28.7 cm for 520 and 445 nm and the axial shift comes from the chromatic aberration in the propagating air [30–32]. FWHMs at 520 and 445 nm are 59.4 and 59.4 μm. Our broadband results match well with previous reported references where the invariance of focal spots is derived from the chromatic focus shift and the constant of *λz* under the paraxial approximation [30–33]. In addition, we analyse the focusing efficiency of our LC element. The simulated efficiency is 33.44% while the measured efficiency is 24.21% at 638 nm. The simulated efficiency is lower than the pure-binary-phase lens because the near-field pattern brings some energy loss. And the discrepancy between the simulated and measured efficiency may be attributed to the fabrication and measurement errors. The focusing efficiencies for 520 and 445 nm are further lower than that for 638 nm because the coefficient of (*t*_u_−*t*_v_) is highest at 638 nm in our fabrication and it decreases when the wavelength deviates from 638 nm. All the above experimental results show that our proposed element has good broadband performance when it works as a focusing lens and a near-field displayer.

**Figure 3:**
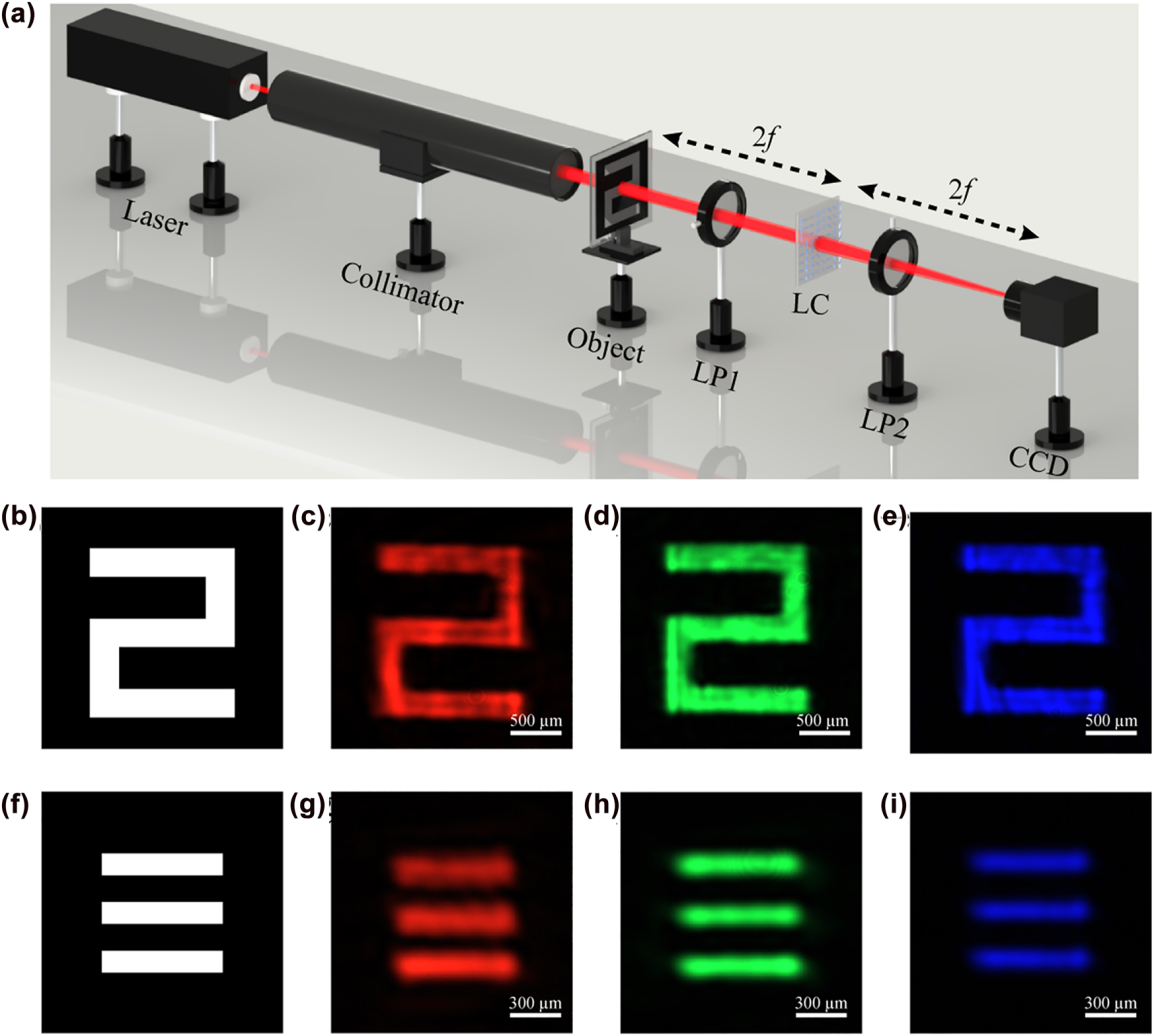
Imaging performance of the multifunctional LC element. (a) Schematic illustration of the imaging setup. (b) and (f) Are target patterns, (c–e) and (g–i) are imaging results of the LC element under the imaging setup at wavelengths of 638 nm, 520 nm, and 445 nm.

To characterize the imaging performance of our LC element, two objects (one is an Arabic numeral (“2”) and the other consists of three lines with the width and separation of 88 and 176 μm) is put at a distance of 2*f* from the sample, as shown in Figure 3(a). Two objects are clearly observed under the red illumination in Figure 3(c) and Figure 3(g). In addition, the imaging results under green and blue illumination have the similar quality and contrast, which indicates that the LC element possesses excellent broadband imaging performance. Furthermore, our proposed element can be applied in the field of optical anti-counterfeiting and security identification technologies. The orthogonal-polarization optical path and surface pattern are regarded as security keys. If the element does not work in the orthogonal-polarization optical path, the correct surface pattern cannot be directly observed by the naked eye, optical microscope or even laser light source, and the focusing function will also be invalid.

### 2.3 A bifocal LC element through spatial multiplexing

**Figure 4:**
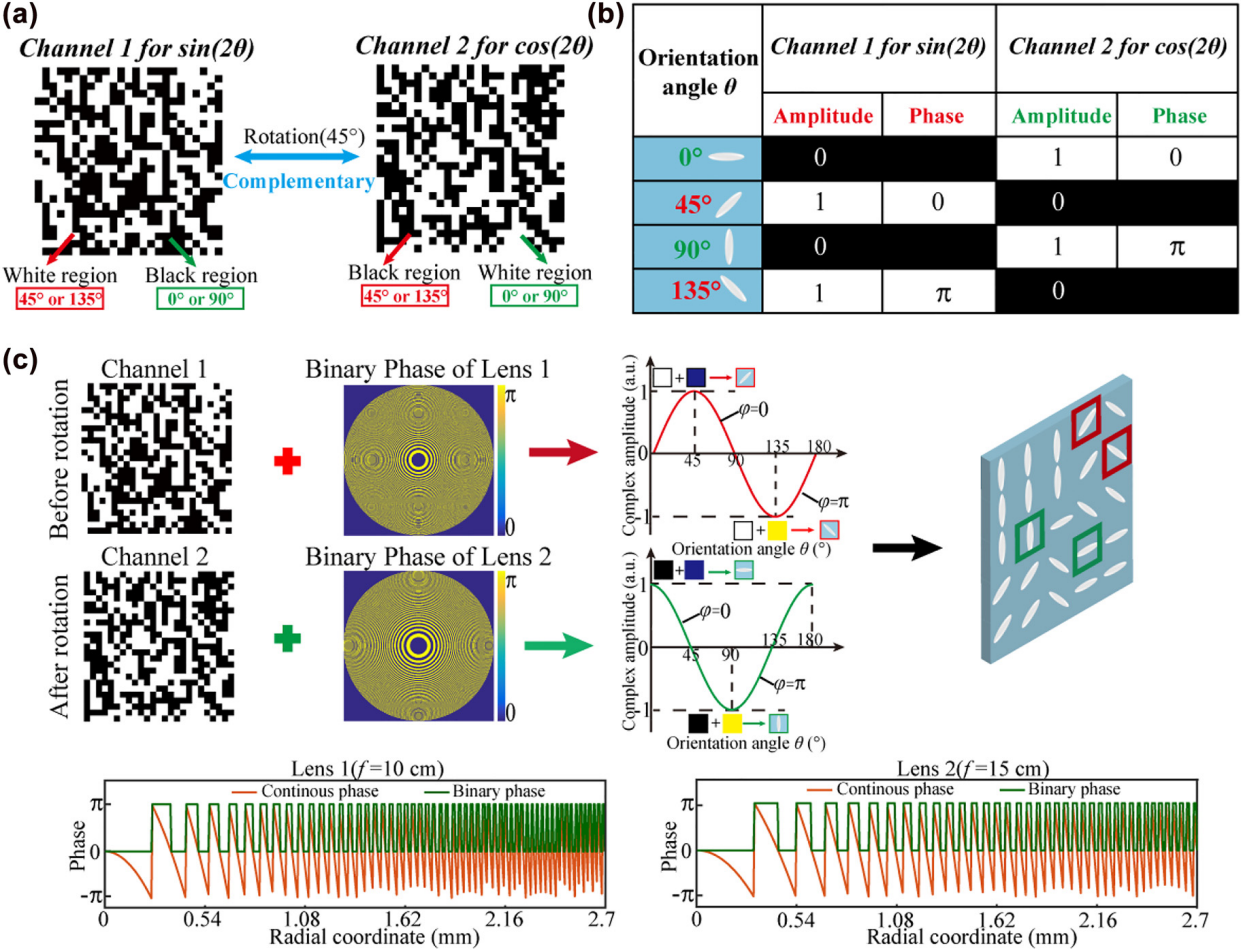
Design flowchart of the spatial multiplexed LC element. (a) White regions in Channel 1 contain LC orientations of 45° and 135° while black regions contain LC orientations of 0° and 90°. Light modulation of sin(2*θ*) changes to cos(2*θ*) if further rotating the sample with 45°. (b) Light modulation under sin(2*θ*) and cos(2*θ*) for four orientations of 0°, 45°, 90° and 135°. Once the light modulation follows sin(2*θ*), the white regions in Channel 1 would provide the modulation of 1 and −1 while the dark regions provide the modulation of 0. If further rotating the sample with 45°, the light modulation follows cos(2*θ*), thus white regions in Channel 1 would provide the modulation of 0 while black regions provide the modulation of 1 and −1, i.e., the complementary pattern would be generated and it is named as Channel 2. (c) Whole design can be regarded as two independent processes in white and black regions. White regions in Channel 1 are optimized to contain orientations of 45° and 135°, while black regions in Channel 1 or white regions in Channel 2 are optimized to contain orientations of 0° and 90°.

As we mentioned earlier, the cross-polarized component of the output light will change from sine to cosine variation when the sample is rotated by 45° and a complementary surface pattern can be observed. Therefore, there exist independent regions under the light modulation of cosine variation to encode an extra binary phase profile. In order to sufficiently utilize these regions and enrich the element function, these regions can be designed as another focusing lens with a different focal length while the near-field pattern is the complementary image. As proof of concept, a spatial multiplexed LC element is designed as a bifocal lens while two complementary near-field patterns can be generated if rotating the sample. The design flowchart of the spatial multiplexed LC element is illustrated in Figure 4. As shown in Figure 4(a), the target pattern (left side) with binary intensity is divided into white and black regions. Before rotation, the complex amplitude modulation follows the relationship of sin(2*θ*), and the white regions of the target pattern correspond to the transmittance regions of the near-field pattern. The orientations of 45° and 135° provide the amplitude of 1 and −1 (the phase difference is *π*) while orientations of 0° and 90° provide the amplitude of 0. After rotating the sample by 45°, the complex amplitude modulation changes from sin(2*θ*) to cos(2*θ*), and the black regions of the target pattern correspond to the transmittance regions of the near-field pattern. The orientations of 0° and 90° provide the amplitude of 1 and −1 (the phase difference is *π*) and the orientations of 45° and 135° provide the amplitude of 0. Therefore, four orientations of 0°, 45°, 90°, and 135° are selected as candidates, and a complementary near-field pattern can be obtained after the rotation of the sample. By combining the target pattern and the desired phase distribution of the bifocal lens, the orientations of LC molecule can be determined, as shown in Figure 4(c).

**Figure 5:**
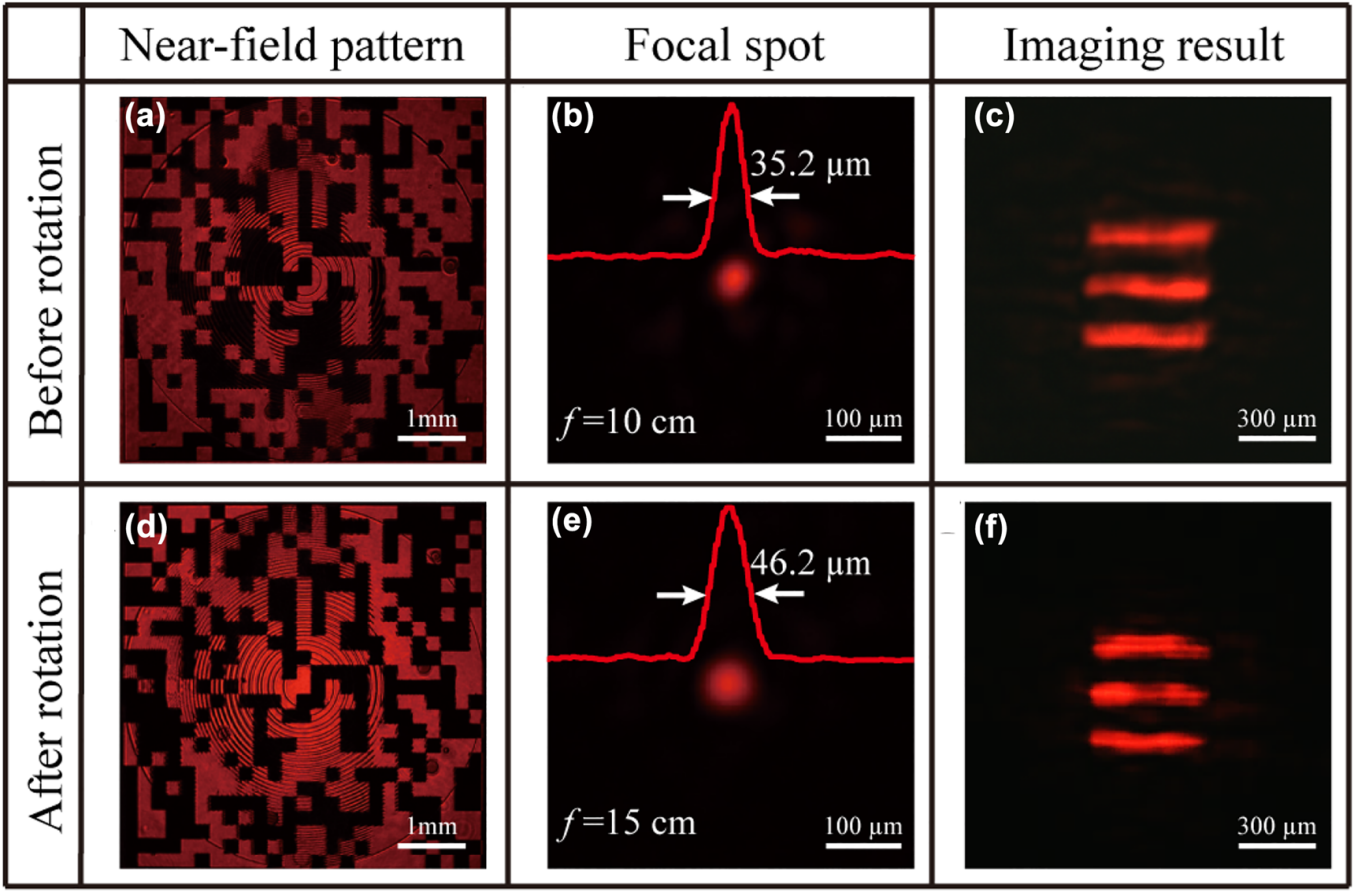
Experimental results of the spatial multiplexed LC element. (a–f) Are near-field patterns, focal spots and imaging results before and after rotation at the wavelength of 638 nm, respectively.

As shown in Figure 5(a) and (b), the near-field pattern of a “random grid” is clearly observed, the focal spot is formed at the plane of *z* = 10 cm under the incident wavelength of 638 nm. After rotating the sample with the angle of 45°, the complementary near-field pattern is displayed, another focal spot is generated at the plane of *z* = 15 cm, as illustrated in Figure 5(d) and (e). The FWHMs of two focal spots are 35.2 and 46.2 μm, respectively (the iris with 2 mm diameter is also used before the sample for a better focusing behavior). Likewise, we choose the object consisting of three lines as the target object to demonstrate the imaging capability of the bifocal element. Figure 5(c) and (f) show the imaging results at two focal planes under red light illumination. The results show that the element has good imaging capabilities at both focal planes. The proposed spatial multiplexing multifunctional element only requires a simple rotation to achieve a fast lens-zoom function. In addition, the proposed LC element is suitable for application to ultracompact optical systems due to the millimeter size. We believe that it will have great potential on compact devices such as smartphones, and will help to further shrink augmented reality (AR) and virtual reality (VR) devices.

## 3 Discussion and conclusion

In the above, the numerical aperture of our proposed LC elements is very small in experiment. The main reason is that a shorter focusing length means that the phase (0 or *π*) changes faster when the position is far from the element center. The faster phase change will cause some fabrication errors, such as the orientation of LC molecules in each pixel cannot be precisely guaranteed by current photo-alignment technology, especially in adjacent areas, where the alignment of LC molecules may be random or gradual due to the intermolecular force, which causes the inhomogeneity in near-field patterns. As shown in Figure 2(a), the regions without the black near-field pattern only contain two LC orientations (45° and 135°). And the intensity in these regions should be equal in theory. However, there are some dark lines between the areas containing orientations of 45° and adjacent areas containing orientations of 135°. It means that the LC orientations in these dark lines may have a gradual orientation change from 45° to 135°. In this case, due to the fast phase change in the adjacent areas, some dark lines may appear and bring the inhomogeneity.

In summary, we demonstrate a multifunctional LC element, which can manipulate the amplitude and phase profiles of the incident light, to work under the dual modes for simultaneous surface displaying and binary-phase light in focus. In experiments, surface displaying patterns and focal spots are clearly observed at the preset planes, which agree well with our designs. Since the light modulation is solely dependent on the orientation of the LC molecule, our element exhibits broadband performance and work well with good imaging capability under a broadband spectrum. Furthermore, a spatial multiplexing technology is introduced to improve the device functionalities. By rotating the sample at two different angles (the angle different is 45°), one can design a bifocal lens with two different focal lengths that can be constructed. The proposed multifunctional LC elements with the advantages of easy fabrication, mature technology, low cost, and large size, may find potential applications in optical fields such as information encryption, storage, security identification, and VR/AR display.

## Supporting information

See Supplementary Material for the supporting content.

## Supplementary Material

Supplementary Material Details
