# Supplementary material for: Planar liquid crystal optics for simultaneously surface displaying and diffraction-limited focusing
Source: Nanophotonics. 2022 Aug 30;11(19):4455–63. doi: 10.1515/nanoph-2022-0410 (PMC11501460; doi:10.1515/nanoph-2022-0410)
Supplement: Supplementary file 1 — Supplementary Material Details [file j_nanoph-2022-0410_suppl.docx]

**Supporting Information**

**Planar liquid crystal optics for simultaneously surface displaying and** **diffraction-limited focusing**

Zhenglong Shao, ^†^ Xin Xie,^§^Yingjie Zhou, ^†^ Xiaohu Zhang,^#^ Wenjuan Du^‡^, Fan Fan^†,^*,Dongliang Tang^†,^*

^†^Key Laboratory for Micro/Nano Optoelectronic Devices of Ministry of Education & Hunan Provincial Key Laboratory of Low-Dimensional Structural Physics and Devices, School of Physics and Electronics, Hunan University, Changsha 410082, China

^§^Key Laboratory of Light Field Manipulation and Information Acquisition, Ministry of Industry and Information Technology, and Shaanxi Key Laboratory of Optical Information Technology, School of Physical Science and Technology, Northwestern Polytechnical University, Xi’an 710129, China

^#^Key Laboratory of Optoelectronic Technology and Systems of the Education Ministry of China, Chongqing University, Chongqing 400044, China

^‡^School of Physics and Optoelectronics, Xiangtan University, Xiangtan 411105, People’s Republic of China

Corresponding Author

*E-mail: ffan@hnu.edu.cn; dltang@hnu.edu.cn

S1. Sample fabrication

The fabrications of the designed multifunctional elements are based on a standard photoalignment technology through using a digital micro-mirror device (DMD), as shown in Figure S1. In order to ensure high-quality samples, the entire preparation process should be kept in a dust-free environment. First, the glass substrate with the thickness of 1.1 mm is completely cleaned by ultrasonic cleaning, sufficient heating, UV-light exposing and blowing with compressed air. Second, sulfonated azo dye (SD1, 0.5%) is dissolved in dimethylformamide (DMF, 99.5%), and the solution is spin-coated on glass substrate to generate an evenly distributed orientation layer. Third, a DMD with different linear polarization states is used to ensure that the SD1 orientations are consistent with the design. Fourth, a solution of LC materials (OS1C-H1 from XAGIC Co.) is dropped on SD1 layer and spin uniformly to form a thin film, where the LC orientations are the same as the SD1 orientations. Here, the LC thickness can be adjusted to making *t_u_*=1 and *t_v_*=-1 at 638 nm, which is also named as half-wave condition. Finally, the LC element is exposed under the unpolarized light with a wavelength of 365 nm for solidifying.


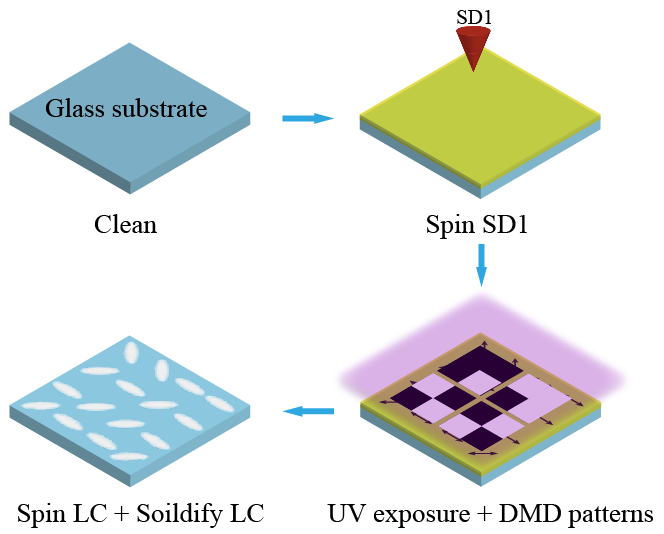


**Figure S1.** The fabrication flow of a LC element with through a standard photoalignment technology and DMD.

S2. Samples with different near-field patterns


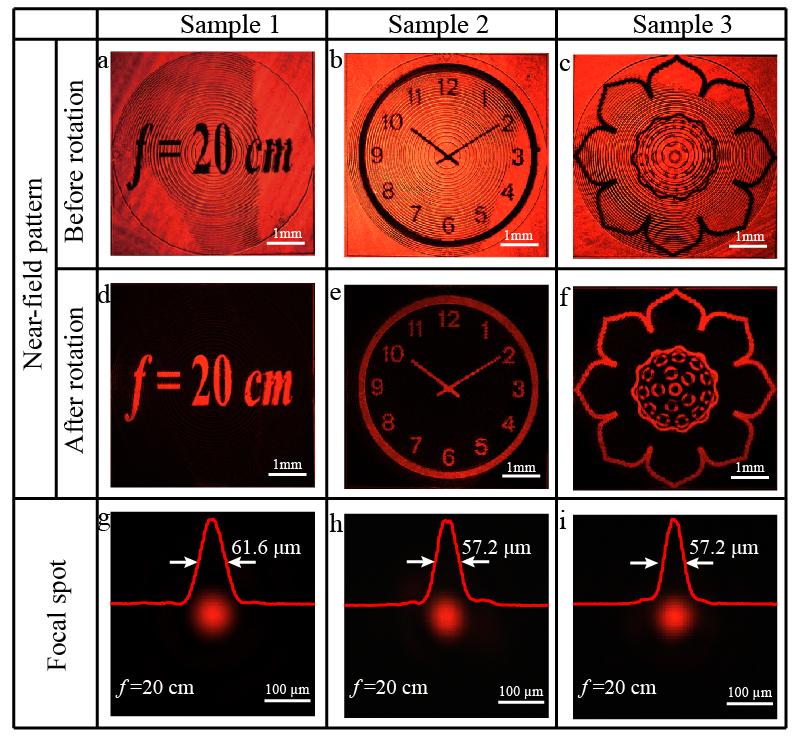


**Figure S2**. Experimental results of three LC elements with different near-field patterns at the wavelength of 638 nm.

S3. The influence of the near-field pattern

In order to better illustrate the diffraction effect caused by the surface display pattern, we fabricate another sample which has no surface display pattern. The diffraction effect of the surface display pattern can be reduced by changing the size of the iris, which is put at the front of the sample. As presented in Figure S3, we measure the focusing performances of two samples through different sizes of the iris at wavelength of 638 nm. We can find that the sample without surface display pattern has good focusing performance through changing the iris diameter, while the sample with surface display pattern has some background noises and the focus deformation. However, the influence can be decreased by reducing the iris diameter. To achieve a better imaging behavior, the iris with the diameter of 2 mm is used in our imaging experiments.


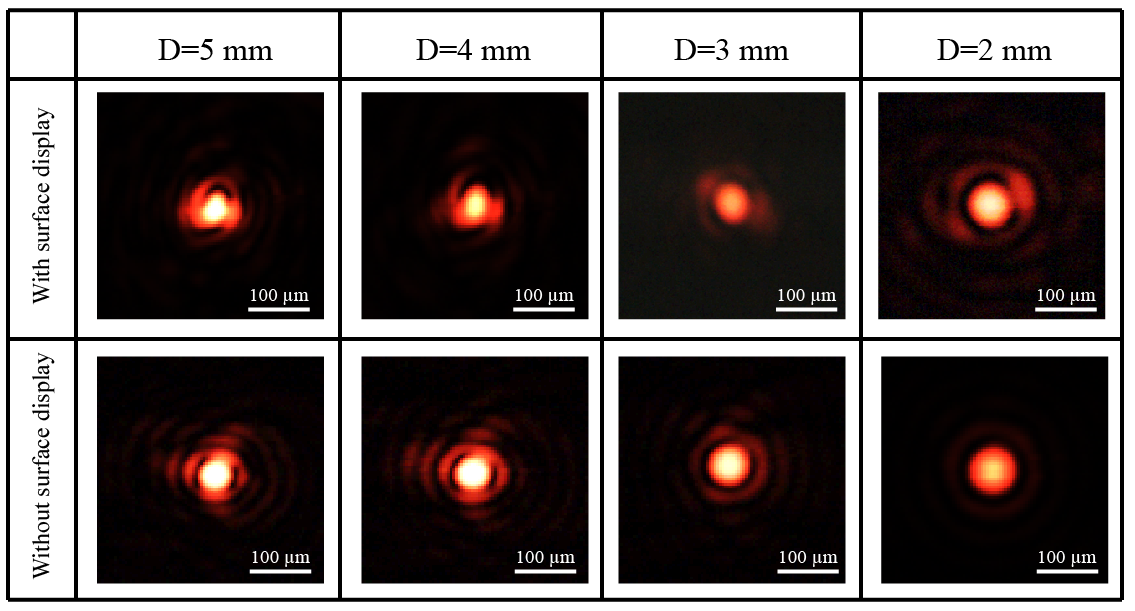


**Figure S3.** Light distributions at the focal plane of two samples through changing the iris diameter, which is put at the front of the sample.
